# Results of a systematic review and meta-analysis of early studies on ivermectin in SARS-CoV-2 infection

**DOI:** 10.1007/s11357-023-00756-y

**Published:** 2023-03-07

**Authors:** Zsuzsanna Ragó, Barbara Tóth, Ágnes Szalenko-Tőkés, Zsolt Bella, Fanni Dembrovszky, Nelli Farkas, Szabolcs Kiss, Péter Hegyi, Mária Matuz, Noémi Tóth, Imre Hegedüs, Domokos Máthé, Dezső Csupor

**Affiliations:** 1 Doctoral School of Clinical Medicine, University of Szeged, Szeged, Hungary; 2 Institute of Pharmacognosy, Faculty of Pharmacy, University of Szeged, Szeged, Hungary; 3 NOÉ Health Care Centre, Szeged, Hungary; 4 Institute for Translational Medicine, Szentágothai Research Centre, Medical School, University of Pécs, Pécs, Hungary; 5 Centre for Translational Medicine, Semmelweis University, Budapest, Hungary; 6 Institute of Bioanalysis, Medical School, University of Pécs, Pécs, Hungary; 7 Division of Pancreatic Diseases, Heart and Vascular Center, Semmelweis University, Budapest, Hungary; 8 Institute of Clinical Pharmacy, Faculty of Pharmacy, University of Szeged, Szeged, Hungary; 9 Department of Biophysics and Radiation Biology, Semmelweis University, Budapest, Hungary; 10 In Vivo Imaging ACF, Hungarian Centre of Excellence for Molecular Medicine (HCEMM), Szeged, Hungary

**Keywords:** Ivermectin, SARS-CoV-2, COVID-19, Meta-analysis, Systematic review

## Abstract

Ivermectin, an antiparasitic drug, has been repurposed for COVID-19 treatment during the SARS-CoV-2 pandemic. Although its antiviral efficacy was confirmed early in vitro and in preclinical studies, its clinical efficacy remained ambiguous. Our purpose was to assess the efficacy of ivermectin in terms of time to viral clearance based on the meta-analysis of available clinical trials at the closing date of the data search period, one year after the start of the pandemic. This meta-analysis was reported by following the PRISMA guidelines and by using the PICO format for formulating the question. The study protocol was registered on PROSPERO. Embase, MEDLINE (via PubMed), Cochrane Central Register of Controlled Trials (CENTRAL), bioRvix, and medRvix were searched for human studies of patients receiving ivermectin therapy with control groups. No language or publication status restrictions were applied. The search ended on 1/31/2021 exactly one year after WHO declared the public health emergency on novel coronavirus. The meta-analysis of three trials involving 382 patients revealed that the mean time to viral clearance was 5.74 days shorter in case of ivermectin treatment compared to the control groups [WMD = −5.74, 95% CI (−11.1, −0.39), *p* = 0.036]. Ivermectin has significantly reduced the time to viral clearance in mild to moderate COVID-19 diseases compared to control groups. However, more eligible studies are needed for analysis to increase the quality of evidence of ivermectin use in COVID-19.

**Supplementary Information:**

The online version contains supplementary material available at 10.1007/s11357-023-00756-y.

## Introduction

COVID-19 disease caused by the novel coronavirus SARS-CoV-2 has rapidly spread worldwide since December 2019, evoking the most devastating pandemic in the twenty-first century. In the first pandemic period, prevention was limited to social distancing and other measures (e.g., wearing a mask, strict hygienic regulations, social distancing). In contrast, the pharmacotherapy of infected patients was relied upon off-label use of some medicines. So far, more than 440 medications have been tried out in the treatment of COVID-19; however, their efficacy is not supported by unequivocal clinical evidence [1]. One of the most widely used and studied drug is ivermectin which is registered to treat lymphatic filariasis, onchocerciasis, and several other parasitic and viral diseases [2].

Several publications support the potential therapeutic use of ivermectin at the molecular and cellular level in COVID-19. Based on a docking study, ivermectin was supposed to be capable of decelerating the viral spread within the human body by binding SARS-CoV-2 and its ACE2 receptor on target cell at two binding sites in the extracellular phase [at position 91 (leucine) of virus spike protein and at position 378 (histidine) of ACE2 molecule] [3]. The prevention of infection may be related to other activities as well as Wagstaff et al. confirmed that ivermectin inhibits the function of importin α/β (Imp α/β) [4]. Moreover, an in silico study revealed that ivermectin, along with other macrocyclic lactone drugs, can block the RNA-dependent RNA polymerase (RdRP) function by preventing the attachment of the RNA template to the enzyme and can also block the RNA elongation by forming H-bonds with two amino acids (Cys622 and Asp760) [5]. Furthermore, ivermectin can dock on the viral-specific RNA helicase, by which action the virus can form only biologically inactive virions [6]. Ivermectin dimers act as ionophores and can transport antiviral zinc ions through the membranes into the cytoplasm from both the intercellular space and the zinc reservoir of the endoplasmic reticulum [7]. In the late phase of COVID-19 disease, ivermectin is able to block the SARS-CoV-2–induced STAT3-mediated cytokine storm [8]. Ivermectin has been shown to exert wide immunomodulatory actions in mouse models and also in humans, by affecting the nicotinic acetylcholine receptor subtype 7 as reported by Laing et al. [9]. Another positive immunomodulatory effect on increased Teff/Treg ratio and tumor targeting CD4+ and CD8+ T cell infiltration was reported in breast cancer mouse model based on interactions with the ATP-P2X4-P2X7 purinergic receptor axis [10]. In children continuously treated for 14 days with a dose of 1000 μg/kg [11] as part of a mixed chemotherapy salvage regimen of acute myeloid leukemia, no toxic effects but stable disease or clinical remission were recorded.

In preclinical in vivo animal studies, ivermectin decreased viral load and improved symptoms in a mouse coronavirus infection with the mouse hepatitis virus (MHV-I) [12]. A preclinical study of ivermectin in a hamster model of SARS-CoV-2 infection [13] proved that ivermectin attenuated clinical scores and symptoms, as well as lung inflammation at 4 days postinfection, after a single subcutaneous dose of 400 μg/kg administered at infection. The symptom-attenuating effects were largely related to the interferon I-III axis, cholinergic and glutamatergic neurotransmitter decrease, and adenylate cyclase gene regulation in this hamster model, somewhat resembling that of corticosteroids and IL-6 antagonists [13].

Based on the robust in vitro antiviral action, the antiviral effects of this compound are under investigation in numerous ongoing studies [14]. However, caution should be taken when translating the in vitro activity to therapeutic efficacy. The first in vitro study of ivermectin on SARS-CoV-2–infected cell lines reported that the compound at a concentration of 5 μM inhibited viral replication within 48 h [15]. However, in this experiment, ivermectin was used at a higher concentration than was previously postulated to be achievable by the typical therapeutic dosage of 150–400 μg/kg. Even in five- or tenfold orally applied concentrations, ivermectin has not shown an increase in adverse effects in human pharmacokinetic studies [16]. Long-standing and widespread use of ivermectin to date has been associated only with infrequent and mostly mild adverse events; severe side effects (encephalopathy) were experienced only in patients co-infected with Loa-Loa [17]. It has been suggested that the polymorphism of the MDR-1 transporter might be related to central nervous system toxicity of ivermectin administration [18]. However, to date, 3.7 billion ivermectin tablet intake has been registered in the VigiBase, whereas only two casualties have been reported as severe neurological adverse events leading to death [18]. Moreover, a recent meta-analysis showed no difference in the severity of the adverse events between standard (up to 400 μg/kg) and higher doses of ivermectin [17].

The number of clinical trials assessing the efficacy of ivermectin in COVID-19 is relatively high [18], while the use of ivermectin has not been accepted in official international organization guidelines, although it is in widespread official use in thirteen countries in Africa, Asia, and Latin-America, with altogether forty-one countries adopting its use to a certain degree in COVID-19 therapy. The European Medicines Agency and the US Food and Drug Administration advised for the use of ivermectin in randomized clinical trials [19, 20]. According to the statement of MSD (the originator of ivermectin) early in the pandemic, there had been no meaningful evidence for the clinical efficacy of ivermectin in patients with COVID-19 disease [21].

Human studies with ivermectin are heterogeneous. Early clinical trials involved diverse study populations treated in different phases of the disease. Defined daily doses, treatment durations, and endpoints are not or just hardly comparable in these trials. In the beginning of the pandemic, efficacy of ivermectin could be assessed based on only case series and retrospective studies; later, randomized controlled trials have been launched.

Our purpose was to evaluate the efficacy of ivermectin in terms of time to viral clearance based on the meta-analysis of available clinical trials in the early period of a year between 30/1/2020 and 31/1/2021.

## Materials and methods

The meta-analysis was performed according to the Preferred Reporting Items for Systematic Reviews and Meta-Analyses (PRISMA—http://prisma-statement.org/PRISMAStatement/PRISMAStatement.aspx) reporting guidance, and it was registered in the International Prospective Register of Systematic Reviews (PROSPERO, registration number CRD42021253185).

The following PICO (patients, intervention, comparison, outcome) format was applied: P: PCR confirmed COVID-19 infected patients; I: ivermectin alone or in combination with standard care or in combination with other drugs; C: standard care or therapy without ivermectin; and O: days required for viral clearance.

For the purpose of continued information of the field, we also include a scoping review activity of ivermectin-related publications on clinical trial results up to 31 Oct 2022, when the formalization of our manuscript was finished. Study reports were retrieved in English in this case, using ClinicalTrials.gov, Google Scholar, and PubMed and searching for ivermectin application reports in clinical trials with emphasis on relevance to our original PICO: early application, mild-moderate disease, and viral clearance*.* We include the results of this overview outside the formal results reporting, in Table 4. in the “Discussion” part.

**Table 1:** Characteristics of the studies included in the quantitative analysis

Study	Country	Design	P (patients)	I (intervention)	C (comparator)	O (outcome)
Khan [29]	Bangladesh	Retrospective, cohort	PCR confirmed COVID-19 patients (*n* = 248)	12-mg ivermectin (single dose) plus standard care	Standard care (as required and included antipyretics for fever, anti-histamines for cough, and antibiotics to control secondary infection)	- Time required for virological clearance - Disease progression (develop pneumonia to severe respiratory distress) - Duration of hospital stays, and - Mortality rate
Ahmed [28]	Bangladesh	double-blinded randomized controlled trial	Hospitalized COVID-19 patients (*n* = 72)	Group A: 12-mg ivermectin daily for 5 days	Group B: 12-mg ivermectin and 200-mg doxycycline on day 1, followed by 100 mg every 12 h for the next 4 days) Group C: placebo	Primary endpoints: - Time required for virological clearance - Remission of fever and cough within 7 days Secondary outcomes: - Failure to maintain an SpO_2_ > 93% despite oxygenation - Days on oxygen support - The duration of hospitalization - All-cause mortality
Babalola [27]	Nigeria	double-blinded randomized controlled trial	PCR-proven COVID-19 positive patients (*n* = 62)	Group A: Standard care plus ivermectin 6 mg every 84 h, twice a week, for 2 weeks Group B: Standard care plus ivermectin 12 mg every 84 h, twice a week, for 2 weeks	Group C: Standard care plus lopinavir/ritonavir daily for 2 weeks	- Time required for virological clearance

### Information sources and search strategy

The systematic literature search included in our meta-analysis was conducted until 31 January, 2021, in Embase, MEDLINE (via PubMed), Cochrane Central Register of Controlled Trials (CENTRAL), bioRvix, and medRvix by using the following search terms: ((“covid 19”) OR (“Wuhan virus”) OR (“coronavirus”) OR (“2019 nCoV”) OR (“SARS-CoV-2”)) AND (ivermectin). To increase the yield of relevant articles, ClinicalTrials.gov was also searched using the above search terms [(ivermectin | ((“covid 19”) OR (“Wuhan virus”) OR (“coronavirus”) OR (“2019 nCoV”) OR (“SARS-CoV-2”))]. No language, publication date, or publication status restrictions were applied. The reference lists of all identified articles were inspected. Only publicly available data were analyzed; the authors were not contacted for additional information.

### Eligibility criteria and study selection

Controlled trials evaluating the effects of ivermectin in PCR confirmed COVID-19 patients were included. Abstracts, case series, case reports, and uncontrolled studies not reporting numerical data on efficacy were excluded. For reference management, EndNote 20 was used. After removing duplicates, the remaining records were screened for eligibility based on their titles and then abstracts. The eligibility of the full texts of the resulting records was assessed by two reviewer teams independently. Based on the agreements and disagreements of the selection, Cohen’s Kappas were calculated. Disagreements between reviewer teams were dissolved by discussion, and if needed, a reviewer previously not involved in the selection was consulted.

### Data extraction and synthesis of results

Study characteristics and results were searched by the two review authors independently. The following data items were extracted from the included papers: study design, characteristics of the patient population and sample size, intervention details, type of comparator(s), outcome measures, and overall results. Days required for viral clearance were extracted as primary outcome measure. Discrepancies in extracted data were resolved by discussion between the two review authors.

### Risk of bias

The risk of bias of controlled randomized studies was analyzed using the Cochrane Collaboration tool for which includes the following domains: random sequence generation, allocation concealment, blinding of participants and personnel, blinding of outcome assessment, incomplete outcome data, selective reporting, and other scores of bias. For each domain, studies were judged to have a high (red), unclear (yellow), or low (green) risk of bias (see Supplementary Figs S1and S2). Disagreement was resolved by discussion. Risk of bias figures were prepared by using the RevMan 5 statistical program [22].

The risk of bias in non-randomized studies of interventions (ROBINS-I) tool was used for assessing the risk of bias of the non-randomized interventional studies [23]. Seven different domains were assessed: confounding, selection of participants, classifications of interventions, deviations from intended interventions, missing data, measurement of outcomes, and selection of the reported outcome. In the end, an overall bias assessment was performed. After evaluation, low, moderate, high risk of bias, or no information were indicated with green, yellow, red, and gray, respectively (see Supplementary FigS3).

The two authors (B.T. and F.D.) first assessed the risk of bias within the selected studies independently, and disagreements were resolved by a third investigator (M.M.). Results of the risk of bias assessment were discussed when the limitations of the individual studies were assessed.

### Statistical analyses

For data synthesis, the methods recommended by the working group of the Cochrane Collaboration were used [22]. The extracted data allowed us to perform a meta-analysis, and the calculated effect sizes were visualized in a forest plot.

For binary outcomes, odds ratios (OR) were calculated with 95% confidence intervals. For continuous outcomes, weighted mean differences (WMD) or standardized mean differences (SMD) with 95% confidence intervals were calculated to investigate the differences between the two groups (ivermectin group vs. control group).

A random-effects model of DerSimonian and Laird was used. Heterogeneity was assessed by using Cochrane’s *Q* and the *I*^2^ statistics. Based on Cochrane’s handbook, *I*^2^ = 100% × (*Q*−df)/*Q* represents the magnitude of the heterogeneity (moderate: 30–60%, substantial: 50–90%, considerable: 75–100%). For the meta-analysis, Stata 15 (StataCorp) was used.

### Quality of evidence

The Grading of Recommendations Assessment, Development, and Evaluation (GRADE) was used for estimating the quality of evidence of all outcomes assessed [24].

## Results

### Systematic search and study selection

**Fig. 1:**
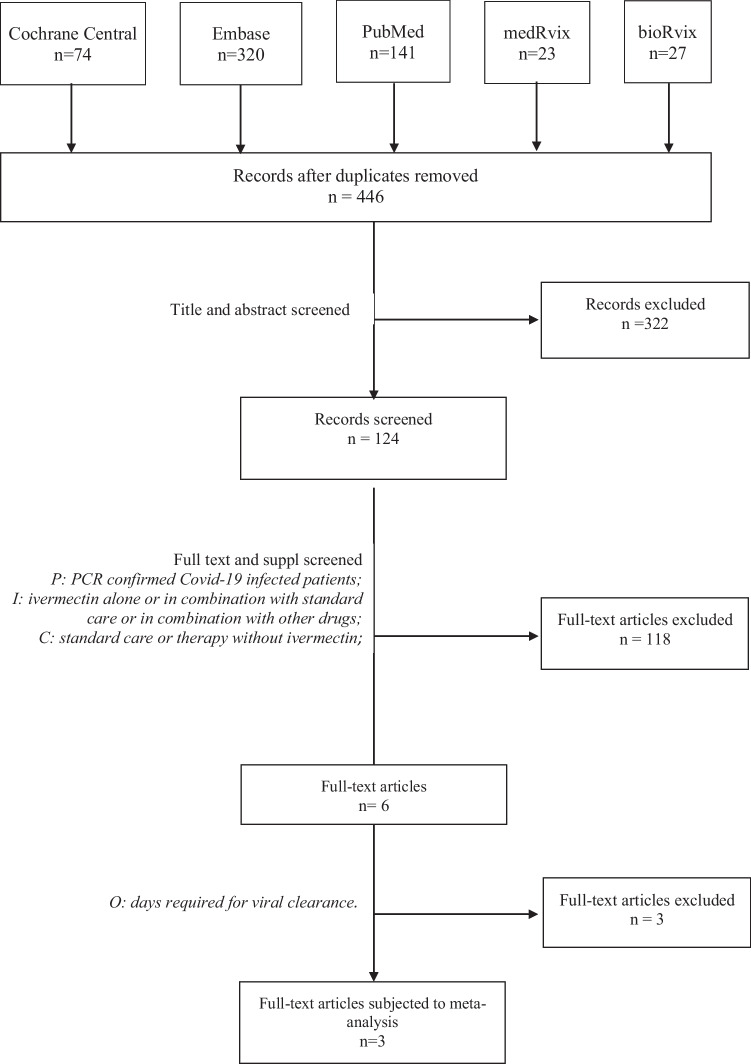
PRISMA flow diagram for identification of relevant studies: 322 records that only reported in vitro or animal experiments, or were review papers were excluded. After screening titles and abstracts, 6 publications were retrieved for qualitative synthesis [25–30], of which three clinical trials were included in the quantitative analysis [27–29] (Table 1)

Using the search key ((“covid 19”) OR (“Wuhan virus”) OR (“coronavirus”) OR (“2019 nCoV”) OR (“SARS-CoV-2”) AND (ivermectin) in Embase, MEDLINE (via PubMed), Cochrane Central Register of Controlled Trials (CENTRAL), bioRvix, and medRvix and removing duplicate results, the search yielded a total of 446 potentially relevant records. The clinical trials included in the meta-analysis were selected according to the PRISMA flow chart presented below (Fig. 1).

### Qualitative analysis of excluded trials

In total, six studies were selected for the qualitative analysis. Of these six studies, three trials were finally not included in the statistical analysis since these trials did not report the outcome of our predefined PICO.

Camprubi et al. compared 13 COVID-19 patients treated with ivermectin (200 μg/kg, single dose) to 13 COVID-19 patients who were not treated with ivermectin in a retrospective study (*n* = 26). No difference was found in any reported clinical and microbiological outcomes (PCR positivity 3–5 days after therapy, clinical improvement 8 days after therapy) [25].

Chaccour et al. assessed the efficacy of a single dose of ivermectin (400 μg/kg) in reducing transmission of SARS-CoV-2 when administered early after disease onset in a placebo-controlled, double-blind RCT (*n* = 24). The primary outcome measure was the proportion of PCR-negative patients at day 7 post-treatment. The viral load and infectivity of each sample were also determined. On day 7, there was no difference in the proportion of PCR positive patients, and the ivermectin group had non-statistically non-significantly lower viral loads at day 4. However, patients in the ivermectin group recovered earlier from hyposmia/anosmia [26].

In these 2 trials, the viral clearance had not been predefined as study outcome.

Okumus et al. conducted an RCT where 66 patients with severe COVID-19 pneumonia were randomized to receive ivermectin (200 μg/kg/day, for 5 days) and a reference treatment protocol hydroxychloroquine, favipiravir, and azithromycin. The control group received only the reference treatment protocol. This study found that adding ivermectin to standard therapy might benefit patients suffering from severe COVID-19 disease (e.g., number of participants with clinical response, oxygen saturation) [30]. In this study, the COVID-19 patients were in severe and not mild or moderate conditions as expected by our PICO.

### Quantitative analysis

Two randomized, controlled, and retrospective studies were included in the meta-analysis.

Babalola et al. conducted a randomized, double blind controlled study involving RT-PCR–proven COVID-19 positive patients in Nigeria. Sixty-two patients were randomized to 6-mg or 12-mg ivermectin regime (given every 84 h for 2 weeks) or lopinavir/ritonavir plus standard care. The period needed for viral negativity was significantly shorter in the two ivermectin groups (6.0 ± 2.9 and for 4.6 ± 3.2 days, respectively, and 5.34 ± 0.07 days for the pooled ivermectin group) versus the control group (9.1 ± 5.2 days). Ivermectin treatment also increased platelet count compared to control, and there was a tendency for increased SPO_2_% in the ivermectin group; however, this difference was not significant. There were no significant changes in hepatic and renal functions, and no adverse drug events were reported spontaneously or in response to inquiry [27].

Ahmed et al. carried out a randomized, double-blind, placebo-controlled trial to determine the duration of viral clearance and safety of ivermectin among adult SARS-CoV-2 patients in Bangladesh. Seventy-two hospitalized patients with mild-moderate disease were assigned to receive ivermectin alone (12 mg once daily for 5 days), or ivermectin in combination with doxycycline (12-mg ivermectin single dose and 200-mg doxycycline on day 1, followed by 100 mg every 12 h for the next 4 days) or placebo. The mean duration to viral clearance was shorter, 9.7 days in the only ivermectin arm (*p* = 0.02), 11.5 days in the ivermectin + doxycycline (*p* = 0.27) arm than in the placebo group (12.7 days). In case of remission of fever, cough, and sore throat, there were no differences between the groups. No adverse effects were recorded during the study [28].

Khan et al. conducted a retrospective study on the data of 325 consecutive patients with SARS-CoV-2 infection in Bangladesh, 115 of whom received ivermectin (single 12-mg tablet within 24 h after hospital admission) plus standard of care (SOC), while 133 received only SOC. The groups were compared in terms of time to SARS-CoV-2 negativity, disease progression, duration of hospital stays, and mortality rate. The time to viral negativity in patients treated with ivermectin was shorter than that in the control group (median 4 vs. 15 days; *p* < 0.001), and the length of hospital stay was also shorter (median 9 vs. 15 days; *p* < 0.001). A total of 9.8% patients developed pneumonia and 1.5% had ischemic stroke in the control group with no such case in the ivermectin-treated group. Significantly, fewer ivermectin-treated patients required oxygen inhalation (9.6% vs. 45.9), developed respiratory distress (2.6% vs. 15.8%), or needed antibiotic therapy (15.7% vs. 60.2%) or intensive care treatment (0.9% vs. 8.3%). There were no side effects reported that can be related to ivermectin use [29].

**Fig. 2:**
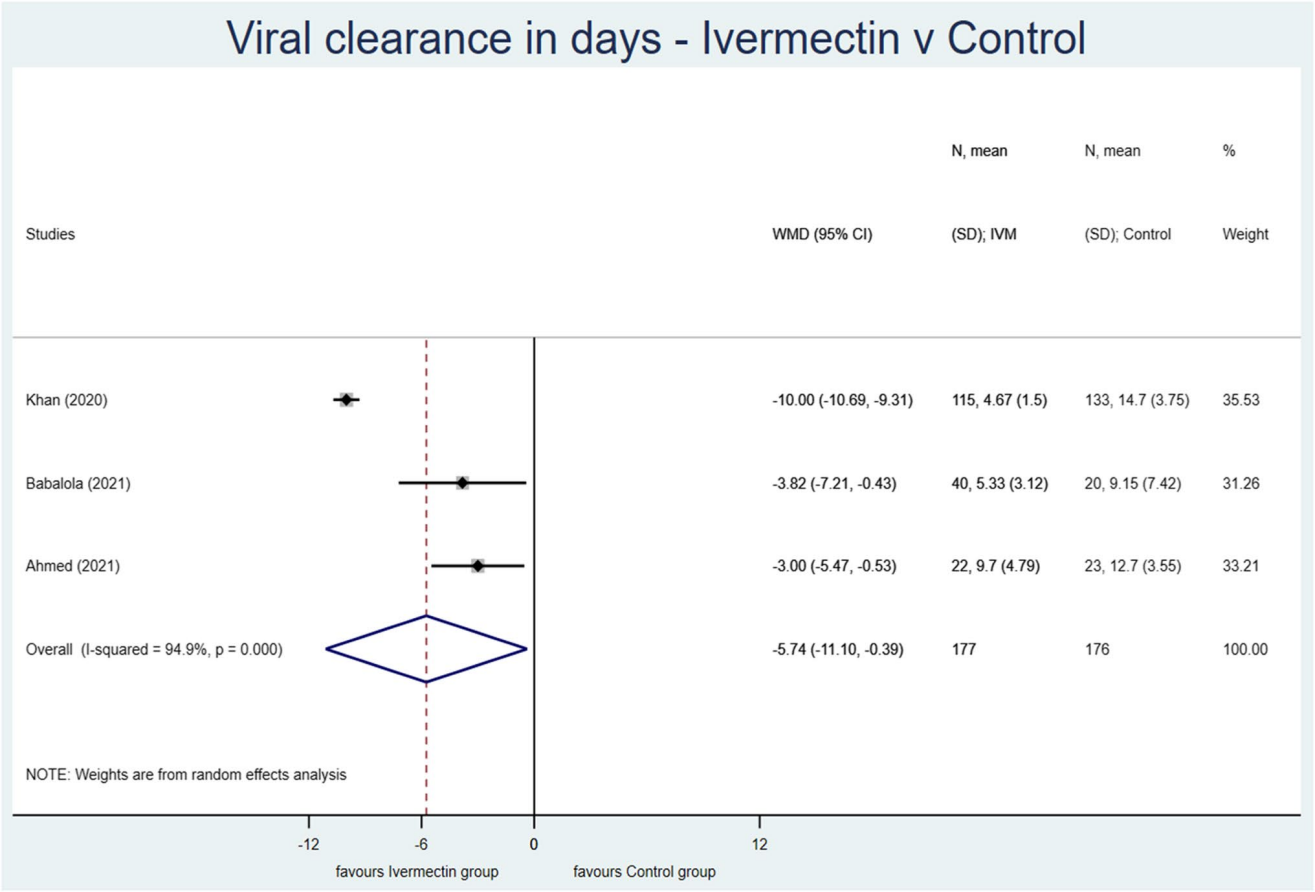
The effect of ivermectin treatment on time to viral clearance

Based on the meta-analysis of the results published in the three analyzed studies [27–29] (Table 2), the mean time to viral clearance was 5.74 days shorter in the case of patients treated with ivermectin than in the controls [*p* = 0.036, WMD = −5.74, 95% CI (−11.1, −0.39)] (Fig. 2).

**Table 2:** Outcomes of the studies included in the present meta-analysis

First author	Ivermectin group						Control group					
	Number of patients	Mean viral clearance (SD) in days	Median viral clearance in days	IQR* min viral clearance in days	IQR max viral clearance in days	95% Confidence interval	Number of patients	Mean viral clearance (SD) in days	Median viral clearance in days	IQR min viral clearance in days	IQR max viral clearance in days	95% Confidence interval (min)
Babalola et al. [27]	40	5.33 (3.12)				4.33–6.32	20	9.15 (7.42)				5.68–12.62
Ahmed et al. [28]	22	9.70 (4.79)				7.80–11.80	23	12.70 (3.55)				11.30–14.20
Khan et al. [29]	115	4.67 (1.50)	4.00	4.00	6.00		133	14.67 (3.75)	15.00	12.00	17.00	

*SD* standard deviation

*Interquartile range

### Risk of bias assessment

Overall, the quality of the randomized trials [27, 28] included in our final quantitative analysis was reckoned to be more disquieting than acceptable, mostly with an unclear risk of bias (see Supplementary Figs S1 and S2).

Both randomized studies showed an unclear risk of selection bias because the authors failed to describe the methods used for randomization in detail [27, 28]. Based on the blinding of the personnel and participants and making the interventions as identical as possible, both studies mentioned above had an unclear risk of performance bias. It was not said in either of the studies whether the intervention and the comparator were identical in size, shape, color, and odor. Furthermore, the authors failed to describe precisely who exactly was blinded, and it was not mentioned in either of the studies whether unblinding occurred before or after data analysis, and the outcome assessment was performed in a blinded manner or not; hence, both studies have an unclear risk of detection bias. Ahmed et al. failed to report on every outcome included in the methods section; therefore, their study was judged to have an unclear risk of reporting bias. The study of Babalola et al. had a low risk of reporting bias, and both randomized controlled studies showed a low risk of attrition bias [27, 28].

The study of Ahmed et al. had an unclear risk of other bias since one of the sponsors manufactures ivermectin-based medication, and it was not indicated whether the sponsor had any influence on the design or the execution of the study [28]. Babalola et al. did not mention any sponsors and conflicts of interest; nevertheless, this does not mean that there were none; therefore, we are uncertain that this study has a low risk of other bias; hence, it was judged as unclear [27].

Khan et al. conducted a retrospective study, and after assessing the risk of bias of the study with the ROBINS-I tool, the overall risk of bias was judged to be low [29]. Moderate bias was assumed only in the case of outcome measurements because assessors might have been aware of the intervention received by study participants. The study showed a low risk of bias in all the six remaining domains (see Supplementary FigS3).

Due to the low number of studies, the presence of publication bias could not be assessed by Egger’s test or funnel plots.

### Grade of evidence

The grade of evidence of our statements was assessed with the GRADE approach (Table 3). To assess the grade of evidence, we considered five downgrading items (i.e., limitations in the design and implementation, indirectness, heterogeneity, imprecision, and publication bias).

**Table 3:** Summary of findings

Population: patients with PCR confirmed COVID-19 infection; intervention: ivermectin; comparison: standard care; and outcome: days required for viral clearance.				
Outcomes	No. of studies included in the qualitative analysis (patients*)	Difference in means (95% confidence interval; *p* value)	Quality of evidence	Comments
Viral clearance	3 (353)	WMD: −5.74 (CI: [−11.1; −0.39], *p* = 0.036)	●○○○ very low	Downgraded for risk of bias and publication bias

*Number of patients

Publication bias is suspected since published evidence includes only a few small trials. Moreover, because of the broad CIs in cases of the trials reported by Babalola et al. and Ahmed et al., imprecision is suspected, and its indirectness is also assumed; hence, the involved patient populations were not homogeneous, and the simultaneously applied therapies are not fully described in the articles [27, 28]. Overall, the finding that ivermectin reduces the time required for viral clearance in COVID-19 patients is supported by very low-quality evidence; i.e., any estimate of effect is very uncertain.

## Discussion

In the light of our findings in this review, it is intriguing to note the quite low doses and short duration of treatment in the referenced clinical studies. To better exploit any putative antiviral effect of ivermectin in planning later studies, first, we propose a re-evaluation of achievable tissue-level virucidal ivermectin concentrations. A dose-escalating phase I study had found no evidence of any harm [14] and a wide safety margin, for per os administration in a dose range from 200 to 2000 μg/kg, three times a week. In the same study, it was also shown that peroral ivermectin administration with a high-fat meal increases the maximal plasma concentration of the same dose more than threefold than in fasted subjects. From the reported data in [14], it can be inferred that even a mg/l plasma concentration is achievable using, e.g., a single 120-mg oral dose with a meal. The effect of meal and even beer to increase ivermectin plasma concentrations have also been reported by others, as well as the absorption-increasing effect of fluid formulation [17]. Schmith et al, in their re-estimation of ivermectin pharmacokinetic tissue distribution models, have called for the re-evaluation of posology in COVID studies because they found that by just using a sufficiently long period of daily, fasted administration with 200 μg/kg, one-fourth of the IC50 reported by Caly et al might even be reached in lung tissue [18]. In the same publication, it was shown that tissue concentrations of ivermectin are expected to be 2–4 times higher than the plasma concentrations. Hence, longer-duration dosing in a daily repeat regimen with a meal and at least using double the approved dose could in fact lead to *tissue* concentrations in the range that have been proven virucidal in vitro. Current clinical and pharmacologic evidence suggests a presumable lack of serious adverse effects from such dose regimens.

Our meta-analysis revealed that the mean time to viral clearance was 5.74 days shorter in patients treated with ivermectin than in those in the control groups. This effect is statistically significant and clinically relevant; however, it should be noted that this result is based on only three clinical studies [27–29].

The therapeutic efficacy of ivermectin has been assessed in several clinical trials [25, 30] and meta-analyses. A meta-analysis assessed the therapeutic potential of ivermectin as an add-on treatment [31], whereas one study focused on its potential role in prophylaxis [32]. A meta-analysis (literature search ended on April 9, 2021) analyzed the effect of ivermectin compared to standard of care or placebo on mortality reported as risk ratio (RR). The results of nine randomized, controlled trials (1788 patients) were meta-analyzed. Ivermectin treatment was associated with decreased mortality (RR 0.39, [95% 0.20–0.74], *p* = 0.004; *I*^2^: 58.2%); this effect was not significant in patients with severe COVID-19 (RR 0.42, *p* = 0.052). The major limitation of this paper was that most of the included studies were preprints. Moreover, in the case of several trials, the sample size was inadequate, and the dosage of ivermectin and the therapy applied in the control group (chloroquine, hydroxychloroquine, favipiravir, standard of care, placebo) was heterogeneous [33].

The primary outcome for the intervention component of the meta-analysis of Bryant et al. included death from any cause and presence of COVID-19 infection. Altogether, 15 trials were (*n* = 2438) meta-analyzed and it was found that ivermectin reduced risk of death compared with no ivermectin (RR 0.38, [95% 0.19–0.73]; *I*^2^ = 49%). The effect was more pronounced in patients with mild to moderate COVID-19 than in severe cases (RR 0.24 vs. 0.51). The limitations of this study are the variability of recruited participants and control treatment regimens [34].

According to a meta-analysis of data obtained involving critically ill patients hospitalized in the intensive care unit (ICU), ivermectin use was associated with lower mortality (OR 0.15, [95% 0.04–0.57]; *p* = 0.005). However, this result was based only on two trials [35].

Besides the scientific papers reporting meta-analyses, the authors of the webpage www.c19early.com are regularly updating the page with clinical data related to ivermectin use since the outbreak of SARS-CoV-2. As of 8/8/2021, all studies show 86%/74%/43% efficacy in prophylaxis/early treatment/late treatment in 61 trials with 23.309 patients.

The strength of our meta-analysis is that we have followed the most recent guidelines during data collection and analysis. One of the limitations of this strategy is the very limited number of eligible studies for meta-analysis over a relatively short period of time. Two of these were carried out in the same country, and the overall number of involved patients is rather low. Moreover, the dosages of ivermectin, the study durations, and patient populations were heterogeneous and the risk of bias was usually unknown due to the poor reporting expectation. Although our study supports the efficacy of ivermectin in decreasing time to viral clearance, further trials and meta-analyses should be carried out to assess its clinical efficacy. In order to maintain actuality, we also considered providing the reader with the recapitulation of studies published up to October 2022 in a tabular format. These trials [35] are summarized in Table 4. Our findings hint that for further meta-analyses dosage of the trial drug, as well as treatment duration must be accounted for and if possible, dissected further with appropriate statistical methodology. It seems apparent from Table 4 that an increased daily dose of ivermectin and longer time of administration might be offering more therapeutic benefits over the maximally reported 5 days of use. It is interesting to note that recent reports such as Kerr’s [45] can direct towards a possible prophylactic use of ivermectin in public health settings as well.

**Table 4:** Scoping tabular review of formal clinical trials involving ivermectin application. Only prospective randomized double-blinded studies were selected.

Source	Publication time	Comparator	Dose, mg/day	Number of treatment days	Patient status	Effect on Primary Endpoints	Conflict of interest reported
Abd-Elsalam et al. [36]	31 May, 2021	Standard protocol of treatment	12	3	Mild, moderate	No significant effect	No
Lopez-Medina et al. [37]	4 March, 2021	Placebo	24	5	Mild	No significant effect	Yes
Vallejos et al. [38]	02 July, 2021	Placebo	24	2	Mild, moderate	No significant effect	No
Mohan et al. [39]	25 August, 2021	Placebo	12 and 24	1	Mild, moderate	No significant effect	No
Samaha et al. [40]	26 May, 2021	Supplements	9 to 16	1	Asymptomatic, prophylaxis	Significant	No
Beltran Gonzalez et al. [41]	3 March, 2022	Hydroxychloroquine and placebo	12 or 18	4	Severe	No significant effect	No
Krolewiecki et al. [42]	18 June, 2021	“No treatment”	30 or 48	5	Mild-to moderate, not in intensive care	Dose-dependent significance	Yes
Biber et al. [43]	2 July, 2022	Placebo	12 and 15	3	Mild	Significant	No
Naggie et al. [44]	21 October, 2022	Placebo	32	3	Mild, moderate	No significant effect	Yes

## Conclusions

Ivermectin has significantly reduced the time to viral clearance in mild to moderate COVID-19 diseases compared to control groups. The mean time to viral clearance was 5.74 days shorter in case of patients treated with ivermectin, which may be a therapeutic advantage. However, the quality of evidence is very low and the results of this meta-analysis do not confirm the therapeutic value of ivermectin in terms of symptom relief, decreased risk of hospitalization, or mortality. Several studies have been finished since our closure date and many are still ongoing to reveal the efficacy of ivermectin in COVID-19 disease which we could not include due to conceptual and outcome analysis differences. New research is needed to analyze recent data and to also evaluate the clinical advantage of ivermectin therapy in SARS-CoV-2 infection.

### Supplementary information

ESM 1: **FigS1** Risk of bias summary: a review of the authors' judgment about each risk of bias item for each included study. **FigS2** Risk of bias graph: a review of the authors' judgment about each risk of bias item presented as percentages across all included studies. **FigS3** Risk of bias assessment of the study of Khan et al.
