# Supplementary material for: Results of a systematic review and meta-analysis of early studies on ivermectin in SARS-CoV-2 infection
Source: GeroScience. 2023 Mar 7;45(4):2179–93. doi: 10.1007/s11357-023-00756-y (PMC9988599; doi:10.1007/s11357-023-00756-y)
Supplement: Supplementary file 1 — FigS1 Risk of bias summary: a review of the authors' judgment about each risk of bias item for each included study. FigS2 Risk of bias graph: a review of the authors' judgment about each risk of bias item presented as percentages across all included studies. FigS3 Risk of bias assessment of the study of Khan et al. [file 11357_2023_756_MOESM1_ESM.docx]

**Supplementary materials**

**
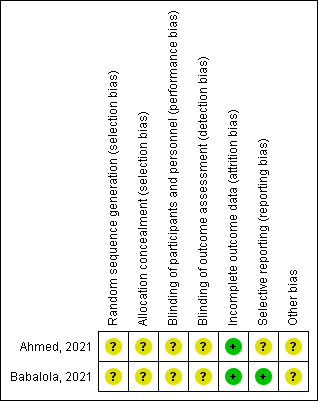
**

**FigS1** Risk of bias summary: a review of the authors' judgment about each risk of bias item for each included study


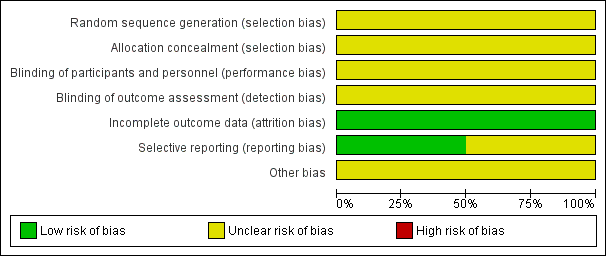


**FigS2** Risk of bias graph: a review of the authors' judgment about each risk of bias item presented as percentages across all included studies


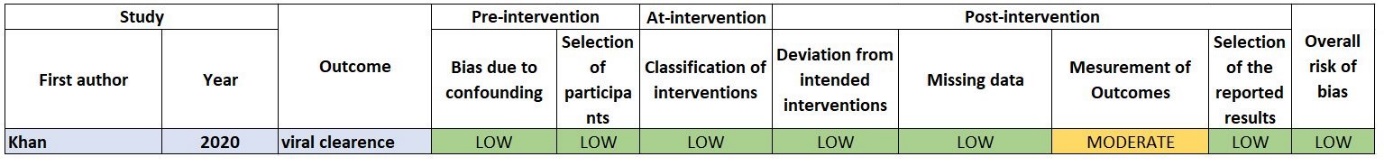


**FigS3** Risk of bias assessment of the study of Khan et al.
